# A Web-Based Prognostic Model for Pediatric Genitourinary Rhabdomyosarcoma: Analysis of Population-Based Cohort With External Validation

**DOI:** 10.3389/fpubh.2022.870187

**Published:** 2022-05-10

**Authors:** Jiayi Li, Yangyue Huang, Yunpeng Li, Pei Liu, Haiyan Cheng, Hongcheng Song, Ning Sun, Mina Ayad Shamil, Weiping Zhang

**Affiliations:** ^1^National Center for Children's Health, Department of Surgical Urology, Beijing Children's Hospital, Capital Medical University, Beijing, China; ^2^National Center for Children's Health, Department of Surgical Oncology, Beijing Children's Hospital, Capital Medical University, Beijing, China; ^3^International School, Capital Medical University, Beijing, China

**Keywords:** Rhabdomyosarcoma, genitourinary, overall survival, nomogram, prognosis, web-based risk calculator

## Abstract

**Background:**

We conduct an analysis of data from the Surveillance, Epidemiology, and End Results (SEER) database, intending to identify prognostic factors of pediatric genitourinary rhabdomyosarcoma (PGU–RMS). Prognostic nomogram and web-based calculator were developed for potential clinical use.

**Methods:**

Data of PGU–RMS patients were extracted from the SEER database as training and internal validation cohort, patients diagnosed as PGU–RMS from 2001 to 2015 in Beijing Children's Hospital were collected as an external validation cohort. We used log-rank tests to seek risk factors on the overall survival (OS) in the overall SEER cohort, tumor site subgroups, radiation subgroups, and metastasis subgroups. The univariable and multivariate Cox regression analyses were applied to establish the prognosis model.

**Results:**

A total of 372 PGU-RMS patients in SEER and 84 patients from our center were included. 1-, 3-, and 5-year OS of the overall SEER cohort were 95.8, 82.1, and 78.8%. Subgroup analysis indicated that tumors located in the prostate/bladder were associated with a worse prognosis than the paratesticular, female genital system, and other sites (*P* < 0.001). Tumors of the T1/T2 stage, without regional lymph node, involvement or metastasis, can benefit from radiotherapy (*P* < 0.05). For patients without metastasis, younger age, T1/T2 stage, and undergoing radiation were associated with better prognosis (*P* < 0.05). The prognosis nomogram was well-calibrated, the concordance index (C-index) for the OS prediction was 0.823, 0.803, and 0.768 in training, internal and external validation cohort, the area under the receiver operating characteristic curve for 3-, and 5-year OS were 0.84, 0.84 in the training cohort, 0.90, 0.84 in internal validation cohort and 0.75, 0.80 in the external validation cohort. Decision curve analysis showed good clinical utility. The predictive performance of the nomogram was higher than the Intergroup Rhabdomyosarcoma Study Group (IRSG) pretreatment stage system based on the comparison of overtime C-index, net reclassification index, and integrated discriminatory index (*P* < 0.001).

**Conclusion:**

A comprehensive analysis of OS for PGU–RMS patients was conducted based on population cohort. The established prognosis nomogram has been fully validated and evaluated, exhibits better performance than the IRSG pretreatment stage system. Furthermore, a web-based risk calculator was developed to optimize clinical decisions.

## Introduction

Rhabdomyosarcoma (RMS) is the most common soft tissue sarcoma in children and ~20–25% of RMS arise from the genitourinary system (1). Because of the special anatomical characteristic, pediatric genitourinary RMS (PGU-RMS) requires more targeted treatment methods to balance the survival and the functions of the involved organs. Multidisciplinary cooperative group trials and multimodal treatment protocols were developed, which have improved the prognosis of RMS over the past few decades (2–4). Indeed, challenges and controversies remained. Despite the fact that radiotherapy has become the primary treatment modality for local control of PGU–RMS, large sample-size randomized controlled trial studies are insufficient to demonstrate the radiation effect on specific patient subgroups (2, 3). Furthermore, a consensus for the treatment of PGU–RMS metastasis (4). Improved survival rates have led to an emphasis on the development of a targeted and facilitated prognostic model of PGU–RMS.

In this study, we conducted a large cohort analysis of data from the Surveillance, Epidemiology, and End Results (SEER) registry to identify the risk factors associated with the prognosis of PGU–RMS. Subgroup analyses were conducted to explore the prognosis of different sites of the tumor, the effect of radiation, and seek subgroup-specific risk factors of children with or without metastasis. Moreover, we established a nomogram for predicting the OS of PGU–RMS and validated it by an external cohort. Finally, a web-based risk calculator was developed for potential clinical use.

## Materials and Methods

### Data Collection

**Figure 1:**
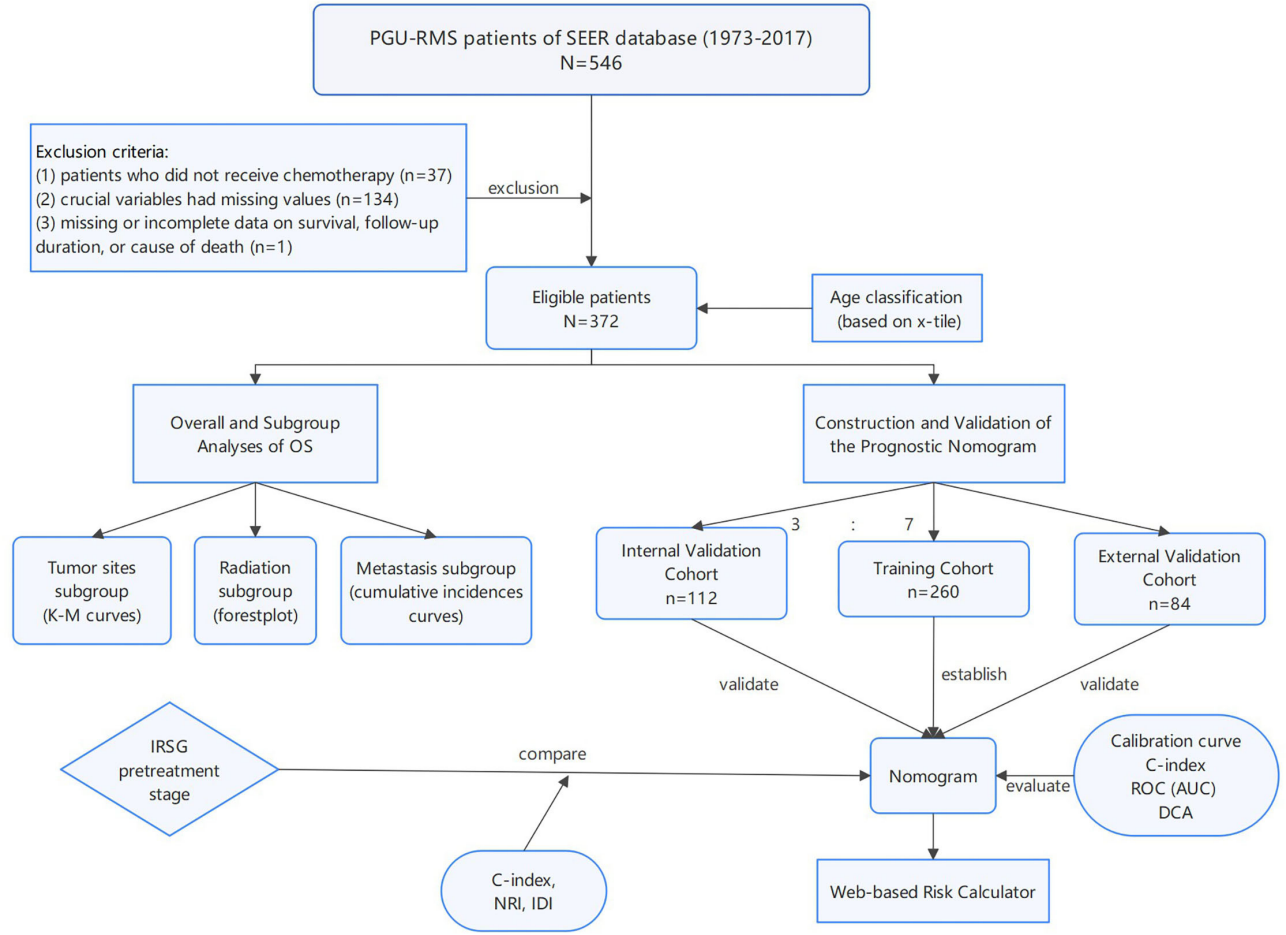
The flowchart including the analysis of survival and the establishment of nomograms to predict OS of patients with PGU-RMS. PGU-RMS, Pediatric genitourinary Rhabdomyosarcoma; OS, overall survival.

The SEER database from 1973 to 2017, which incorporated data from 18 cancer registries and represents ~35% of the American publication (5) was used to identify a cohort of PGU–RMS patients, retrospectively. The study included patients under 20 years old who were diagnosed with PGU–RMS (confirmed by histological diagnosis) as their first primary malignant tumor. The following International Classification of Diseases for Oncology, Third Edition (ICD-O-3), histology codes were extract: 8,900–8,902, 8,910, 8,912, 8,920, and 8,991, and also the Site recode ICD-O-3: C51 (vulva), C52 (vagina), C53 (cervix), C54/55 (uterus), C61 (prostate), C62 (testis), C63 (other male genital organs), C67 (bladder), and C68 (urethra/other urinary system). The exclusion criteria were as follows: (1) patients who did not receive chemotherapy (as the standard therapy of RMS). (2) crucial variables had missing values (tumor size and metastasis status). (3) missing or incomplete data on survival, follow-up duration, or cause of death. After applying the exclusion criteria, the final study contained 372 PGU–RMS patients (Figure 1). For external validation, we extract patients' data following the same inclusion and exclusion criteria from Beijing Children's Hospital, Capital Medical University, National Center for Children's Health.

Information of each patient extracted from the SEER database included age, sex, ethnicity, diagnosis year, primary tumor site, tumor size, T, N, M stages, SEER historic stage, tumor histology, and treatment modality (surgery, radiotherapy). The X-tile program (Yale University, New Haven, Connecticut, USA) was exploited to define the optimal cut-point of age at diagnosis (6), divided patients into three groups which were <14, 14–16, and 17–19 years old (Supplementary Figure 1). The tumor histology variable was dichotomized as alveolar against non-alveolar (including embryonal, spindle cell, pleomorphic, mixed type, and cases not otherwise specified). The SEER historic staging categorizes disease as Localized, Regional, and Distant were clearly defined and adjusted by the SEER program (7). As a curial rating scale, the Intergroup Rhabdomyosarcoma Study Group (IRSG) pretreatment staging system was determined according to the primary site and TNM stage of each patient (8). The corresponding clinical data of the external validation cohort were retrospectively collected from electronic medical records, clinic, and telephone follow-up were used to collect the prognostic information.

### Analysis of Overall Survival

The primary endpoint of this study was OS, which was defined as the survival time computed from the time of diagnosis to the death due to any cause or the time of the last follow-up for patients still alive. The overall analysis of OS was performed by Cox proportional hazards regression models for univariable analysis, of which the interval hazard ratio (HR) and the associated 95% CI were evaluated. Subgroup analysis of OS was compared with log-rank tests, and the results were expressed as a Kaplan-Meier for tumor sites subgroups, forest plot for radiation subgroups, and cumulative incidences curves for metastasis subgroups (Figure 1).

### Construction and Validation of the Nomogram

Children meeting the aforementioned criteria were involved. After simple random sampling, the overall SEER cohort was divided into a training cohort and a validation cohort in a 7:3 split ratio. In the training cohort, univariate and multivariate Cox proportional hazards regression analysis were applied, variables with statistically significant in the univariate analysis were further included in the multivariate regression analysis, and specific variables were selected to build the nomograms and the web-based risk calculators, predicting the OS of patients.

Internal and external validations were applied to evaluate the predictive ability of the established nomogram. Calibration curves were used to demonstrate the reliability, the discrimination was evaluated by concordance index (C-index) and time-dependent receiver operating characteristic (ROC) curve analysis, as well as a clinical utility, was assessed by decision curve analysis (DCA) (9, 10). Furthermore, we compared the predictive performance between the nomogram survival model and the IRSG pretreatment staging system. Over-time C-index curves were conducted to compare the discrimination of the two models, and the net reclassification index (NRI) and integrated discriminatory index (IDI) were calculated to compare the abilities of reclassification and integrated discrimination (11) (Figure 1).

### Statistical Analysis

SEER^*^Stat version 8.3.8 (National Cancer Institute, Bethesda, MD) statistical software was used to extract relevant patients' information. Statistical analysis was all conducted by R software (version 4.0.3, http://www.r-project.org). Continuous data not following normal distribution are presented as median and inter-quartile range, analyzed by the Mann-Whitney U test, while variables between groups were compared using the chi-square test or Fisher's exact test. All the statistical results were reported as two-tailed *p-*values < 0.05 is considered statistical significance.

## Results

### Characteristics of the Patients

A total of 372 PGU–RMS patients from the SEER database were incorporated in the study. The baseline demographic clinical characteristics of the patients are summarized in Table 1. The median age of the overall cohort was 6 years and the optimal cutoff values were confirmed as 13 and 16 years old by the X-tile program (Supplementary Figure 1). Male (*n* = 287, 77.2%) and non-Hispanic (*n* = 287, 77.2%) patients are accounted for a greater proportion of overall cohort. Prostate or bladder, as unfavorable sites of RMS, was less than other sites (29.6 vs. 70.4%). Besides, ~7% of alveolar-type RMS was observed in all patients, retrospectively. As for treatments, most patients underwent surgery (*n* = 309, 83.1%), and approximately half of the patients underwent radiotherapy (*n* = 184, 49.5%). Overall SEER cohort (*n* = 372) was randomly assigned into the training cohort (*n* = 260) and the internal validation cohort (*n* = 112), PGU–RMS patients (*n* = 84) from Beijing Children Hospital were selected as the external validation cohort. The clinicopathological characteristics between these three cohorts was shown in Table 1.

**Table 1:** Characteristics of patients with PGU-RMS in the training cohort and validation cohorts.

	**Training Cohort, *N* = 260**	**Internal Validation Cohort, *N* = 112**	**External Validation Cohort, *N* = 84**
Age (years)	6.0 [2.0, 14.0]	5.5 [2.0, 14.0]	3.04 [1.58;6.00]
**Age classification:**			
~13	167 (64.2%)	74 (66.1%)	78 (92.9%)
14~16	61 (23.5%)	25 (22.3%)	6 (7.14%)
17~	32 (12.3%)	13 (11.6%)	0 (0.00%)
**Sex:**			
Female	56 (21.5%)	29 (25.9%)	16 (19.0%)
Male	204 (78.5%)	83 (74.1%)	68 (81.0%)
**Ethnicity:**			
Hispanic	55 (21.2%)	30 (26.8%)	0 (0.00%)
Non-Hispanic	205 (78.8%)	82 (73.2%)	84 (100%)
**Diagnosis Year:**			
1975–2003	107 (41.2%)	52 (46.4%)	7 (8.33%)
2004–2016	153 (58.8%)	60 (53.6%)	77 (91.7%)
**Tumor Site:**			
Prostate/bladder	77 (29.6%)	33 (29.5%)	48 (57.1%)
Other sites	183 (70.4%)	79 (70.5%)	36 (42.9%)
Tumor size (mm)	60.0 [40.0, 90.0]	55.0 [40.0, 90.0]	56.0 [35.0;85.2]
**T stage:**			
T1	114 (43.8%)	49 (43.8%)	31 (36.9%)
T2	99 (38.1%)	43 (38.4%)	23 (27.4%)
T3	36 (13.8%)	15 (13.4%)	17 (20.2%)
T4	11 (4.23%)	5 (4.46%)	13 (15.5%)
**N stage:**			
Nx/N0	205 (78.8%)	99 (88.4%)	58 (69.9%)
N1	55 (21.2%)	13 (11.6%)	26 (30.1%)
**M stage:**			
M0	205 (78.8%)	82 (73.2%)	75 (89.3%)
M1	55 (21.2%)	30 (26.8%)	9 (10.7%)
**SEER historic stage:**			
Localized	114 (43.8%)	54 (48.2%)	46 (54.8%)
Regional	91 (35.0%)	28 (25.0%)	29 (34.5%)
Distant	55 (21.2%)	30 (26.8%)	9 (10.7%)
**Tumor histology:**			
Alveolar	20 (7.69%)	6 (5.36%)	2 (2.38%)
Non-alveolar	240 (92.3%)	106 (94.6%)	82 (97.6%)
**IRSG pretreatment stage:**			
I	114 (43.8%)	53 (47.3%)	24 (28.6%)
II	72 (27.7%)	27 (24.1%)	37 (44.0%)
III	19 (7.31%)	2 (1.79%)	13 (15.5%)
IV	55 (21.2%)	30 (26.8%)	10 (11.9%)
**Radiation:**			
Yes	136 (52.3%)	48 (42.9%)	30 (35.7%)
No	124 (47.7%)	64 (57.1%)	54 (64.3%)
**Surgery:**			
Yes	216 (83.1%)	93 (83.0%)	84 (100%)
No	44 (16.9%)	19 (17.0%)	0 (0.00%)

### Overall and Subgroup Analysis of OS

As shown in Table 2, 1-, 3-, and 5-year survival of the overall cohort were 95.8, 82.1, and 78.8%. Variables associated with poorer prognosis were older age, primary tumor site of the prostate or bladder, presence of metastasis, higher T stage, SEER historic stage, and IRSG pretreatment stage (*P* < 0.001). However, sex, ethnicity, regional LN involved, and tumor histology did not correlate significantly with patients' OS (*P* > 0.05). Regarding treatment, surgery had significantly improved survival (*P* < 0.001) but radiation did not show a significant impact on OS rates in the overall cohort (*P* = 0.06).

**Table 2:** Overall survival rates of patients with PGU-RMS in SEER cohort.

**Characteristic**	**Patients**		**Overall survival rate (%)**			***P***
	**No**.	**%**	**1-year**	**3-year**	**5-year**	
Total	372	100.0	95.8	82.1	78.8	
**Age classification:**						**<0.001**
~13	241	64.8	96.1	85.8	83.6	
14–16	86	23.1	96.4	80.6	76.6	
17~	45	12.1	93.3	61.4	56.4	
**Sex:**						0.7
Female	85	22.8	96.3	81.7	77.2	
Male	287	77.2	95.7	82.1	78.7	
**Ethnicity:**						0.9
Hispanic	85	22.8	96.2	81.4	78.3	
Non-Hispanic	287	77.2	95.7	82.3	78.9	
**Tumor Site:**						**<0.001**
Bladder/prostate	110	29.6	91.5	68.5	65.0	
Other sites	262	70.4	97.7	87.7	84.0	
**T stage:**						**<0.001**
T1/T2	305	82.0	96.6	84.8	82.3	
T3/T4	67	18.0	92.3	70.2	63.4	
**N stage:**						0.07
Nx/N0	304	81.7	95.9	83.9	80.6	
N1	68	18.3	95.6	74.6	69.6	
**M stage:**						**<0.001**
M0	287	77.2	98.9	92.6	89.5	
M1	85	22.8	83.3	47.6	43.7	
**SEER historic stage:**						**<0.001**
Localized	168	45.2	99.4	96.0	94.4	
Regional	119	32.0	97.4	87.8	82.5	
Distant	85	22.8	83.3	47.6	43.7	
**Tumor histology:**						0.1
Alveolar	26	7.0	96.0	71.4	63.0	
Non-alveolar	346	93.0	95.8	82.9	80.0	
**IRSG pretreatment stage:**						**<0.001**
I	167	44.9	98.7	94.0	90.8	
II	99	26.6	97.8	91.9	86.7	
III	21	5.6	95.2	85.4	76.9	
IV	85	22.8	83.3	47.6	43.7	
**Radiation:**						0.06
Yes	184	49.5	96.7	79.3	74.9	
No	188	50.5	95.0	84.9	82.1	
**Surgery:**						**0.001**
Yes	309	83.1	98.3	85.9	82.0	
No	63	16.9	83.9	63.5	-	

*Bold indicates statistical significance*.

**Figure 2:**
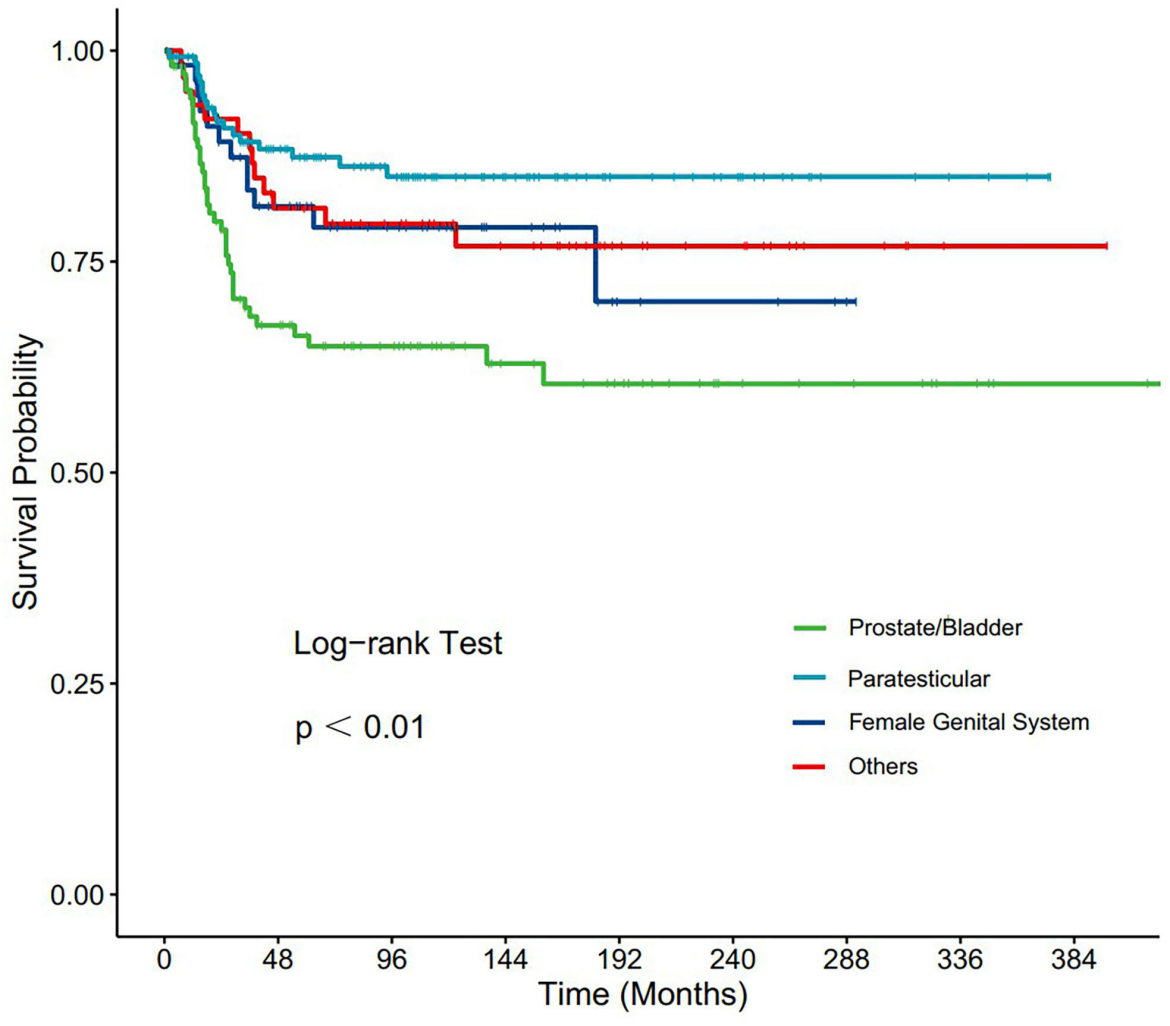
Subgroup analysis of OS for overall SEER cohort; Kaplan-Meier curves for different tumor sites.

**Figure 3:**
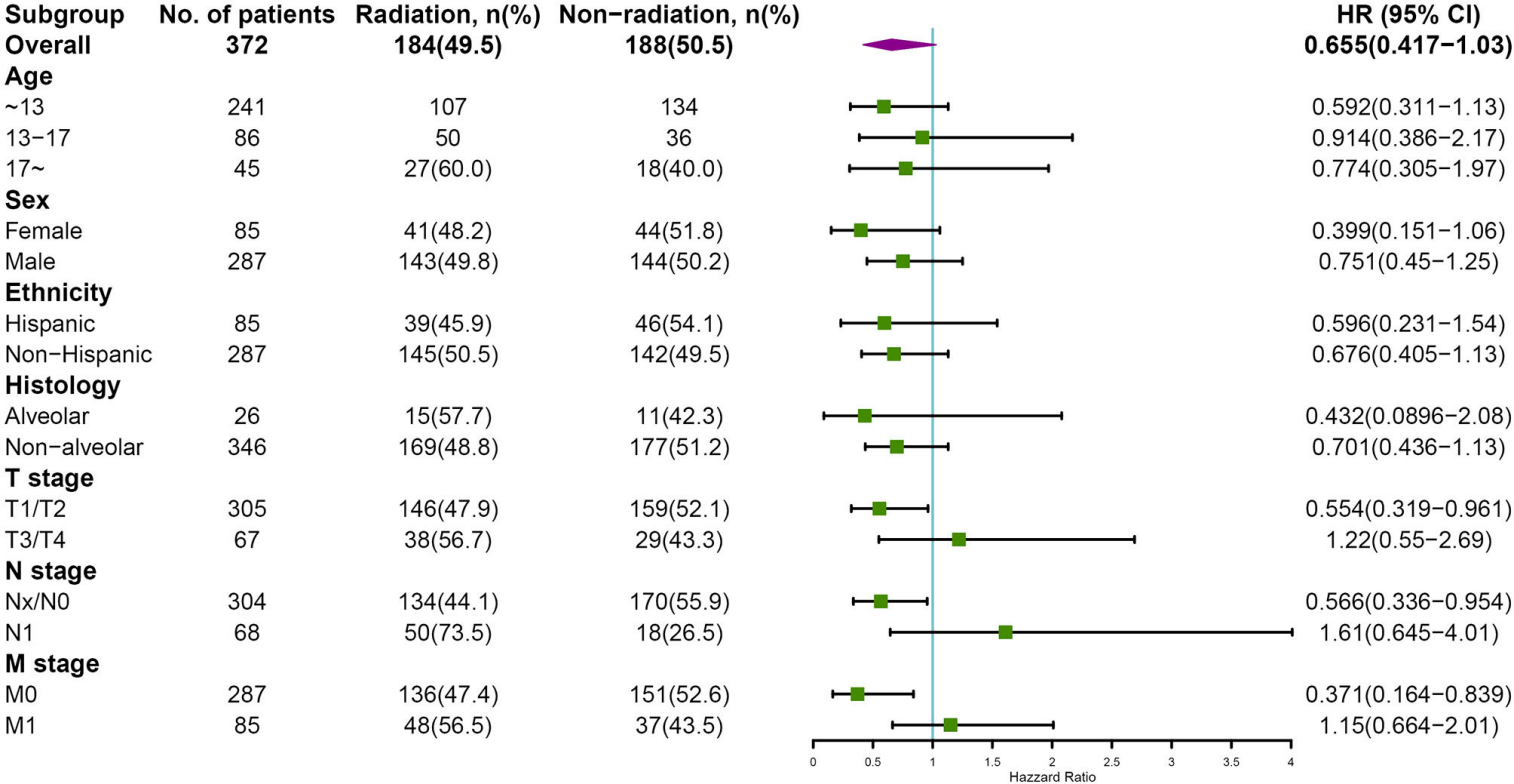
Subgroup analysis of OS for overall SEER cohort; Forest plot of radiation therapy. HR, hazard ratio; 95%CI, 95% confidence interval.

**Figure 4:**
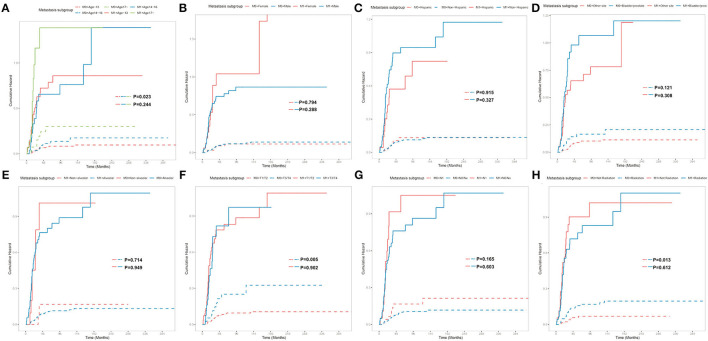
Subgroup analysis of OS for overall SEER cohort; Cumulative incidence curves for metastasis subgroup and patient characteristics: **(A)** Age classification, **(B)** Sex, **(C)** Ethnicity, **(D)** Tumor site, **(E)** Tumor histology, **(F)** T stage, **(G)** N stage, **(H)** Radiation.

Subgroup analyses were conducted to explore the underlying clinical heterogeneity of radiation and metastasis. As shown in Figure 2, tumors located in the prostate/bladder were associated with a worse prognosis than the paratesticular, female genital system, and other sites (*P* <0.001). Figure 3 shows that radiation did not show protective efficacy in the overall cohort. Particular patient subgroups, including T1/T2 stage (HR: 0.554, 95% CI: 0.319–0.961), without regional LN involved (HR: 0.566, 95% CI: 0.336–0.954) or metastasis (HR: 0.371, 95% CI: 0.164–0.839) can benefit from radiotherapy (*P* <0.05). In the subgroup analysis of patients without metastasis, higher survival rates were found in younger patients, T1/T2 stage along with patients' who have received radiation (*P* < 0.05) nevertheless there is no statistically significant difference in the metastasis group (*P* > 0.05). However, the results demonstrated no significance between other subgroups (*P* > 0.05, Figure 4).

### Construction and Validation of the Prognostic Nomogram

**Figure 5:**
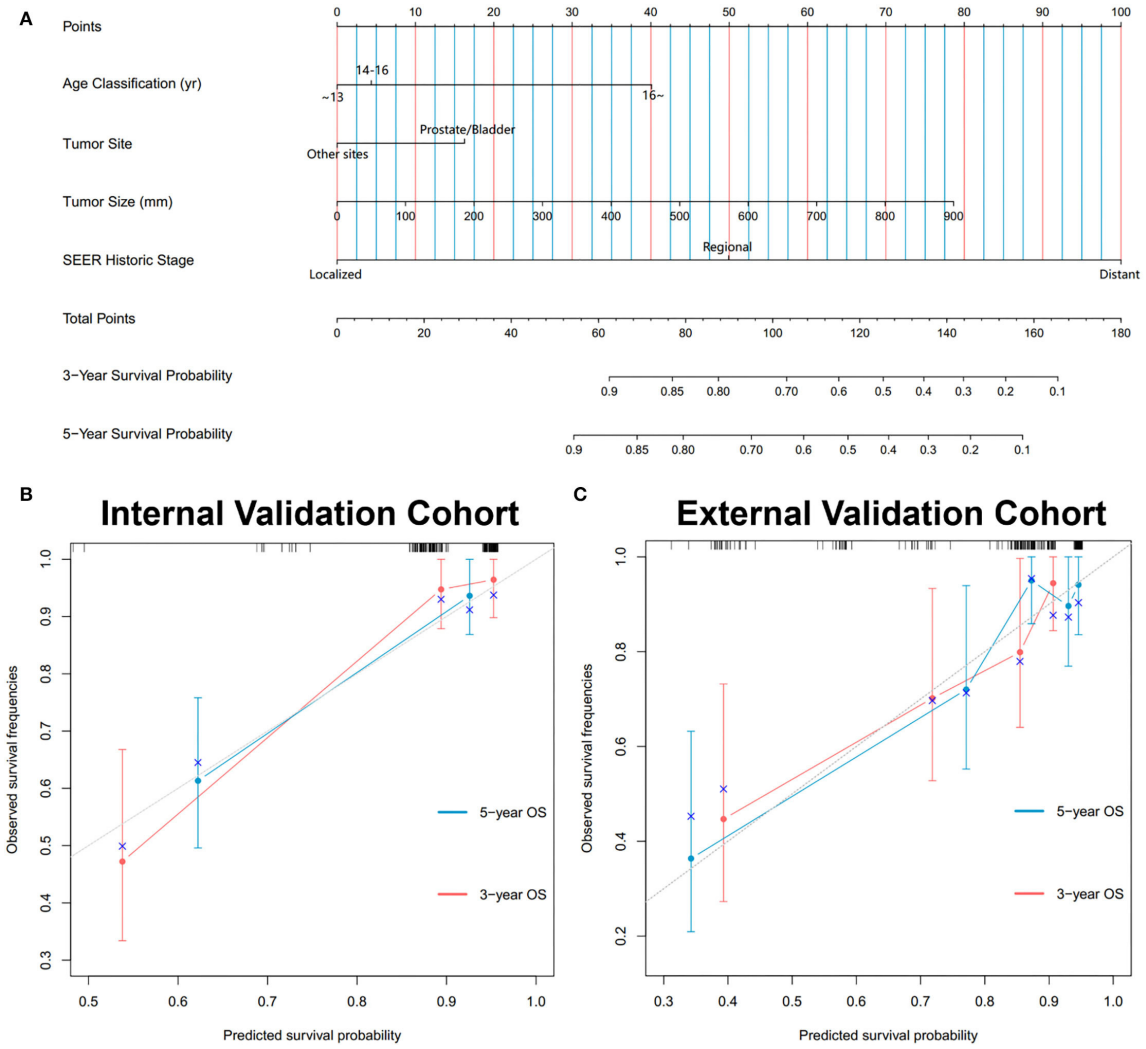
Development and validation of the nomogram predicting OS for PGU-RMS patients. **(A)** Nomograms predicting 3- and 5-year OS. Calibration curves of the nomogram for 3- and 5-year OS prediction in **(B)** internal validation cohort and **(C)** external validation cohort. To use the nomogram, an individual patient's value is located on each variable axis, and a line is drawn upward to determine the number of points received for each variable value. The sum of these numbers is located on the Total Points axis, and a line is drawn downward to the survival axes to determine the likelihood of 3- or 5-year survival. The x-axis and y-axis of the calibration plot represent the nomogram-predicted probability of survival and actual OS probability, respectively. The gray line stands for excellent agreement and the red line stands for the prediction of the nomogram, the redder line overlapping the gray line, the better reliability of the prognosis nomogram.

In the training cohort, a total of 9 factors, including age classification, tumor site, tumor size, TNM stage, SEER historic stage, and radiation, were detected to be statistically associated with the OS of PGU–RMS depending on the univariate Cox proportional hazards regression analysis (*P* < 0.05). Furthermore, we included age classification, tumor site, tumor size, SEER historic stage, and tumor histology in the multivariable regression model. The TNM stage and IRSG pretreatment stage were excluded from the multivariable model because of the collinearity with SEER historic stage. Tumor histology was identified due to the potential clinical significance of OS (Table 3). Multivariate Cox analysis demonstrated that age classification (HR: 1.74, 95% CI: 1.161–2.607), tumor site (HR: 1.855, 95% CI: 1.161–2.607), tumor size (HR: 1.003, 95% CI: 1.0002–1.005), and SEER historic stage (HR: 4.047, 95% CI: 2.594–6.316) were independent risk factors for OS of PGU–RMS, which were used to fit the prognostic model and construct the nomogram (Figure 5).

**Table 3:** Univariable and multivariable Cox proportional hazards regression analysis of OS in PGU-RMS patients for nomogram.

**Characteristic**		**Univariate cox analysis**			**Multivariate cox analysis**		
		**HR**	**95% CI**	**P**	**HR**	**95% CI**	***P***
Age classification	~13/14–16/17~	1.74	(1.25–2.43)	**0.001**	1.74	(1.161–2.607)	0.037^*^
Sex	Female/Male	1.15	(0.576–2.28)	0.699	-	-	NI
Ethnicity	Hispanic/Non-Hispanic	0.96	(0.495–1.86)	0.905	-	-	NI
Tumor Site	Favorable site/Non-Favorable site	2.17	(1.27–3.71)	**0.005**	1.855	(1.161–2.607)	0.049*
Tumor size	mm	1.002	(1.001–1.004)	**0.007**	1.003	(1.0002–1.005)	0.037*
T stage	T1/T2/T3/T4	1.69	(1.29–2.22)	**<0.001**	-	-	NI
N stage:	Nx&N0/N1	0.516	(0.293–0.909)	**0.022**	-	-	NI
M stage:	M0/M1	8.09	(4.67–14.0)	**<0.001**	-	-	NI
SEER historic stage	Localized/Regional/Distant	4.66	(3.08–7.06)	**<0.001**	4.047	(2.594–6.316)	<0.001*
Tumor histology	Alveolar/Non-alveolar	0.579	(0.262–1.28)	0.178	1.065	(0.474–2.393)	0.879
Radiation:	yes/no	2.03	(1.13–3.65)	**0.018**	1.53	(0.842–2.779)	0.163
Surgery:	yes/no	0.564	(0.302–1.05)	0.072	-	-	NI
IRSG stage	I/II/III/IV	2.34	(1.84–2.98)	**<0.001**	-	-	NI

*HR, hazard ratio; CI, confidence interval; NI, not included*.

* *Characteristics included in the nomogram*.

*Bold indicates statistical significance*.

**Figure 6:**
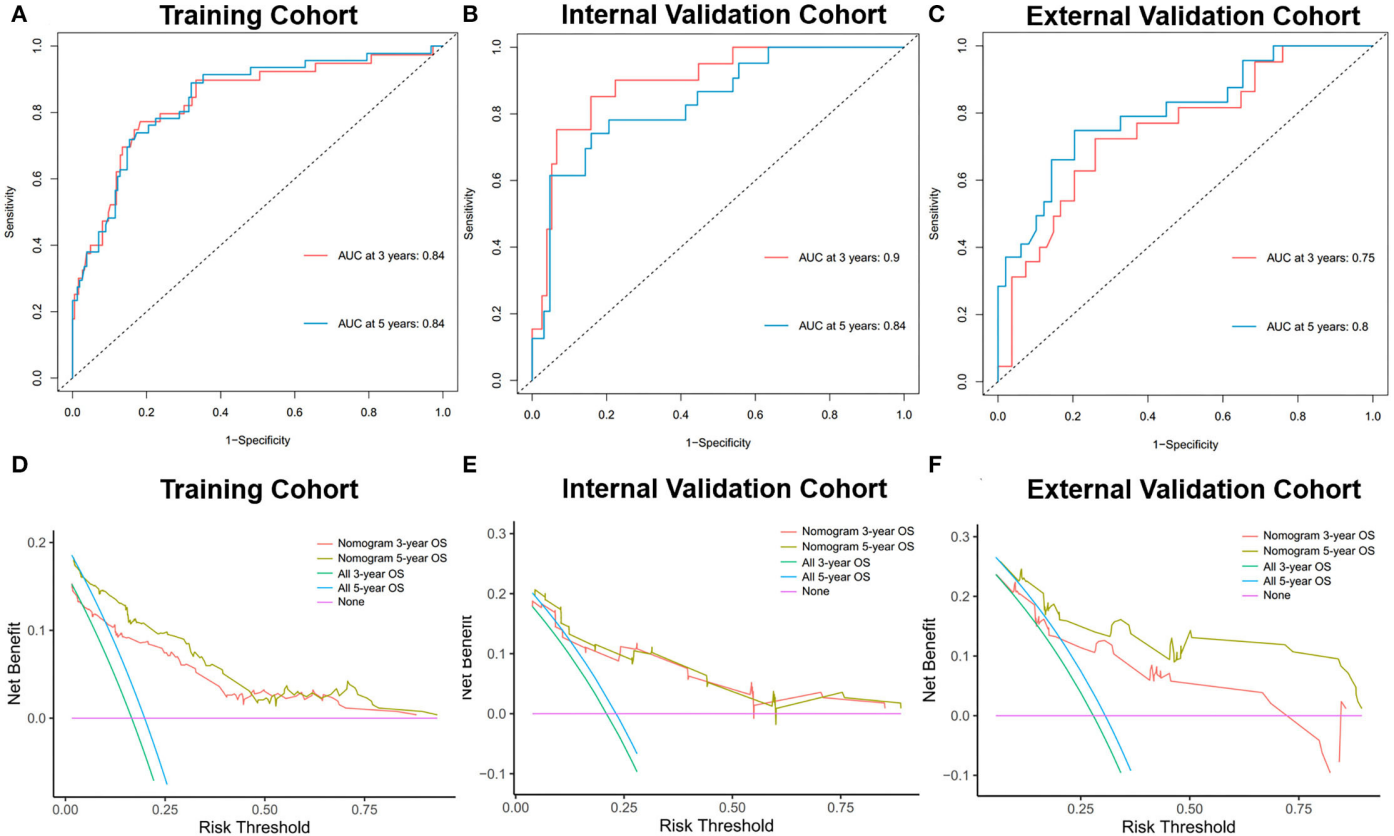
Time-dependent ROC curves of the nomogram predicting 3- and 5-year OS of PGU-RMS patients in **(A)** training cohort, **(B)** internal validation cohort, and **(C)** external validation cohort. AUC values reflect the discrimination performance of the nomograms. The DCA curves of the nomogram predicting 3-, 5-year OS of PGU-RMS patients in **(D)** training cohort, **(E)** internal validation cohort, and **(F)** external validation cohort. The x-axis of DCA curves measures the threshold probability y-axis measures the net benefit. The black line assumes that no patient was correctly predicted and the gray line assumes that all patients have been predicted. ROC, receiver operating characteristic; AUC, area under the receiver operating characteristic curve; DCA, decision curve analysis.

During the evaluation and validation of the prognostic nomogram, the total points of each patient in validation cohorts were calculated based on the established nomogram, then the c-index and calibration curves were derived incorporating the points as an independent variable. As shown in Figure 5, calibration curves revealed an optimal agreement between the predictions estimated of 3- and 5-years OS by the nomogram and actual observations. C-index of the nomogram in training cohort was 0.823 (95% CI: 0.768–0.878), in internal validation cohort was 0.803 (95% CI: 0.795–0.811) and external validation cohort was 0.768 (95% CI: 0.674–0.862). Moreover, time-dependent ROC analysis illustrated the area under the curve (AUC) for OS (training cohort: 3-year OS 0.84, 5-year OS 0.84, internal validation cohort: 3-year OS 0.90, 5-year OS 0.84, external validation cohort: 3-year OS 0.75, 5-year OS 0.80, Figure 6), suggested the relatively ideal discriminative ability of the prognostic nomogram. Finally, DCA curves for 3- and 5-year OS of the nomogram were presented in Figure 6, revealing the model had great clinical utility for PGU–RMS patients.

**Figure 7:**
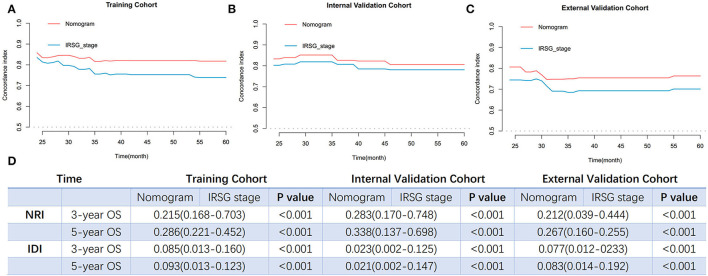
The comparisons of predict performance between the prognosis nomogram and the IRSG pretreatment stage system. Overtime C-index curves for predicting OS of PGU-RMS patients with nomogram and IRSG pretreatment stage system in **(A)** training cohort, **(B)** internal validation cohort, and **(C)** external validation cohort. **(D)** The NRI and IDI values between two models in the training cohort and validation cohorts. NRI values were calculated to compare reclassification ability and IDI values were calculated to compare integrated discrimination ability. C-index, concordance index; NRI, reclassification index; IDI, integrated discriminatory index.

To further validate and compare the predictive performance of the established nomogram and IRSG pretreatment stage system of OS for PGU–RMS, we plotted the over-time c-index curves, additional NRI and IDI values were calculated. As shown in Figure 7, higher c-indexes of the nomogram were screened than which of the IRSG pretreatment stage system overtime in both training cohort (0.823 [0.768–0.878] vs. 0.754 [0.687–0.821]), internal validation cohort (0.803 [0.795–0.811] vs. 0.781 [0.701–0.861]) and external validation cohort (0.768 [0.674–0.862] vs. 0.706 [0.602–0.810]). The NRI and IDI at 3-, and 5-year OS were consistent with the results of over-time c-index curves (*P* <0.001), indicating the prognostic nomogram with better reclassification and integrated discrimination abilities.

### Web-Based Application for OS Prediction

As various methods have been used to demonstrate the superiority of the prognostic model of PGU–RMS, we further developed a web-based risk calculator to help clinical prediction and promote individualized evaluation (https://lijiayi.shinyapps.io/PGU-RMS_riskcalculator/). Individual predictions with a relative CI are calculated using the predict function, displaying either graphically as an interactive plot in the Graphical Summary tab or as a table in the Numerical Summary tab. The table of model output is also available in the Model Summary tab. It should be noted that this online calculator was developed only for research and it should not be used clinically until further validations.

## Discussion

With the development and recognition of PGU–RMS' complexities, optimal treatment strategies are constantly evolving, and survival rates have substantially improved over the decades (12). Nevertheless, strategies for individualized and meticulous prognosis assessment need to be further explored. Notably, PGU–RMS cases occur sporadically, with an overall incidence of about 4.5% of patients per million individuals under 20 years old (1). The inability to collect a sufficient number of patients disables researchers from drawing robust conclusions. In this study, we sought to analyze the independent risk factors for OS, establish and evaluate the prognostic model at a population level, which could minimize the bias of insufficient sample size. Finally, we developed a web-based risk calculator for OS of PGU–RMS, translating research outputs for potential clinical use.

In this study, the 1-, 3-, and 5-year OS rates of PGU–RMS were identified to be 95.8, 82.1, and 78.8%, which were consistent with the previous studies (13, 14). The results of the Cox proportional hazards regression analysis demonstrated age classification, tumor site, TNM stage, SEER historic stage, IRSG pretreatment stage, and surgery as important predictors of PGU–RMS survival. Consensus has been reached in previous studies that age is a curial prognostic factor for pediatric RMS (15–17). Joshi et al. (18) argued that even if disease characteristics were different among patients of different ages, these features cannot provide a satisfactory explanation for the worse outcome for children ages <1 or >9 years old. In this study, we used X-tile software to determine the optimal cut-off point of age at diagnosis associated with the prognosis, poor OS rates were observed with the increase of age classifications, which is consistent with previous conclusions. However, patients aged <13 years, who accounted for 64.8% of the overall cohort, were unevenly distributed, which may potentially lead to bias. As for the tumor sites, according to the historical Children's Oncology Group risk stratification, bladder, or prostate sites are considered unfavorable, presenting a relatively low survival rate, therefore, cannot be treated as low risk (19–21). Our multivariate analysis of survival showed that the survival rate of bladder or prostate tumors was 1.86 times lower compared to other favorable sites, which is in accordance with other studies. Besides, previous studies have always separated different tumor sites in their analysis because of the different treatment modalities toward them. Thus, we analyzed and presented the Kaplan-Meier curve of different tumor sites, the result revealed that tumors located in the prostate/bladder were associated with a worse prognosis than the paratesticular, female genital system, and other sites.

Recent studies have tried to explore the role of adjuvant radiotherapy in the treatment of PUG–RMS (22, 23). It was revealed that radiation was associated with the modest survival benefit, especially in organ preservation in the genitourinary system. Researchers augured that there may be opportunities to minimize the side effect of radiation while maintaining acceptable survival (8, 16, 24). In this study, the presence of metastasis has a strong influence on the OS rate, which may dilute the statistical significance of radiation (*P* = 0.06). Therefore, we conducted two subgroups analyses to adjust confounding factors. The result showed that radiation was effective against selected patients—smaller tumor burden (T1/T2 stage), evidenced by negative regional LN and the absence of metastasis. However, Perez et al. (16) argued that radiation was associated with a survival advantage only for metastasis patients with RMS. In the metastasis subgroup analysis, we found that the younger patients and smaller tumors were associated with better survival for non-metastasis patients but the difference was not statistically significant for metastasis patients, which were partly congruent as expected and by earlier observations (19, 25). Indeed, the standard for radiotherapy and the treatment strategies for metastasis of PGU–RMS patients were evolving rapidly and the OS has been improving over the decades, which may cause an inevitable time-related bias of treatment over a 45-year time interval.

With the development of more aggressive therapeutic regimens for PGU–RMS, more and more attention has been paid to individual differences in treatment methods and prognosis predictions. To the best of our knowledge, this is the first study to build the nomogram for prognostication of PGU–RMS, based on the large cohort size and long-term follow-up period, and quantify the probability of OS on an individual basis. The established prognosis nomogram was validated by both internal and external cohorts, showing relatively strong predictive performance. The well-fitted calibration plots indicated an agreement between predicted and observed outcomes. The discrimination of the nomogram in predicting OS was excellent with relatively high c-indexes and AUC values of time-dependent ROC analysis. Furthermore, the result of DCA demonstrated the nomogram can assist clinical decisions to improve patient outcomes. Previous studies demonstrated that histology/fusion status is usually a major prognostic factor of RMS, however, we were unable to improve the performance of the model even after several attempts. We believe that there are several reasons for this result. First, the different molecular features of different histology may cause a similar prognosis, the fusion gene status irrespective of histology is a critical factor in the risk stratification of RMS. Studies revealed that the overall and event-free survival, frequency of metastases, and distribution of site at initial presentation were not significantly different between fusion-negative alveolar and embryonal RMS (26). However, we were unable to extract genetic and fusion information from the SEER database. Second, only 20 alveolar patients were identified in our training cohort, the sample size may limit the reliability of results. In future studies, we will continue to collect more patient information prospectively, incorporating the histology and gene-fusion status to improve the performance of the nomogram.

The tumor-node-metastasis (TNM) stage system, initially proposed in 1953, was the gold standard for prognostication in oncology for decades (27). Furthermore, the IRSG pretreatment stage system combined the TNM system with the primary tumor site, as an essential component of IRSG prognostic stratification of RMS (28). However, the IRSG pretreatment stage is a system with a finite number, unable to incorporate continuous variables for tumor size, lymphatic nodes, or metastasis, complicating the determination of an individual patient's prognosis. In this study, we constructed a targeted prognosis model of PGU–RMS, including age, tumor site, continuous tumor size, and SEER historic stage as predictive variables, developed a nomogram as pictorial representations. According to the established prognosis model, patients with different characteristics would be assigned different scores to estimate overall survival times, no matter if they were classified as the same TNM stage or IRSG pretreatment stage. As for the comparison of the two models, higher overtime c-indexes of the nomogram than the IRSG pretreatment stage system indicated better discrimination. In addition, the results of NRI and IDI values showed an improvement of more than 20% in reclassification ability and ~10% in integrated discrimination ability of the prognosis nomogram. Given that the IRSG pretreatment stage cannot be directly obtained from the SEER database, as far as we know, this is the first study that compared the IRSG pretreatment stage system with the established nomogram of RMS. Finally, as the superiority of the established prognostic nomogram, we built a web-based calculator for providing accurate and individualized survival prediction in the PGU–RMS patients. We believe the rapid computation through user-friendly digital interfaces, together with increased accuracy, and more easily understood prognoses compared with the conventional staging of the web-based application will aid the clinical decision making.

Nonetheless, limitations should be considered in the study as noted previously. At first, the OS analysis and model established were based on administrative SEER datasets, subjecting to the inherent limitations, including missing data, insufficient detailed data for disease and therapies, assuming that outcomes remain constant over time, which may cause the inevitable biases. Second, the inherent recall biases of a retrospective study would be difficult to avoid. As the conclusions in our study were not completely in accordance with previous studies, the investigations of different treatment modalities in optimizing clinical outcomes in particular patients should be explored, and future studies should extend the model to a higher predict precision and clinical utility.

## Conclusion

We conducted a comprehensive analysis of data from the SEER registry to identify prognostic factors of PGU–RMS. The prognostic nomogram was constructed and fully validated and evaluated, exhibiting better performance than the IRSG pretreatment stage system. In addition, a web-based risk calculator was developed to potentially assist in optimizing the physicians' clinical decisions. We believe our work fulfills our desire for clinically integrated models and our drive toward personalized medicine.

## Data Availability Statement

The raw data supporting the conclusions of this article will be made available by the authors, without undue reservation.

## Ethics Statement

The studies involving human participants were reviewed and approved by Committee of Beijing Children's Hospital, Capital Medical University, National Center for Children's Health. Written informed consent from the participants' legal guardian/next of kin was not required to participate in this study in accordance with the national legislation and the institutional requirements.

## Author Contributions

JL and YH designed the study and analyzed the data. YL collected the data of the external validation cohort. PL and HC revised the article. WZ, NS, and HS supervised the study. MS helped in the grammatical revision of the article. All authors contributed to the article and approved the submitted version.

## Conflict of Interest

The authors declare that the research was conducted in the absence of any commercial or financial relationships that could be construed as a potential conflict of interest.

## Publisher's Note

All claims expressed in this article are solely those of the authors and do not necessarily represent those of their affiliated organizations, or those of the publisher, the editors and the reviewers. Any product that may be evaluated in this article, or claim that may be made by its manufacturer, is not guaranteed or endorsed by the publisher.

## Supplementary Material

Supplementary Figure 1 The X-tile program was exploited to define the optimal cut-point of age at diagnosis. **(A)** The graph shows that the optimal cut-point was identified as 13 and 16 years old. **(B)** Distribution histogram of three groups divided by two cut-point in the overall cohort. **(C)** Significant differences between groups were evaluated using Kaplan-Meier's analysis (*P* < 0.05).

## Abbreviations

SEER: Surveillance, Epidemiology and End Results
PGU-RMS: pediatric genitourinary RMS
OS: overall survival
LN: lymph node
RMS: Rhabdomyosarcoma
IRSG: Intergroup Rhabdomyosarcoma Study Group
SIOP: International Society for Pediatric Oncology
ICD-O-3: International Classification of Diseases for Oncology, Third Edition
HR: hazard ratio
CI: confidence interval
C-index: concordance index
ROC: receiver operating characteristic
DCA: decision curve analysis
NRI: net reclassification index
IDI: integrated discriminatory index
TNM: tumor-node-metastasis.
