# Supplementary figures and images for: A Web-Based Prognostic Model for Pediatric Genitourinary Rhabdomyosarcoma: Analysis of Population-Based Cohort With External Validation
Source: Front Public Health. 2022 May 10;10:870187. doi: 10.3389/fpubh.2022.870187 (PMC9127601; doi:10.3389/fpubh.2022.870187)

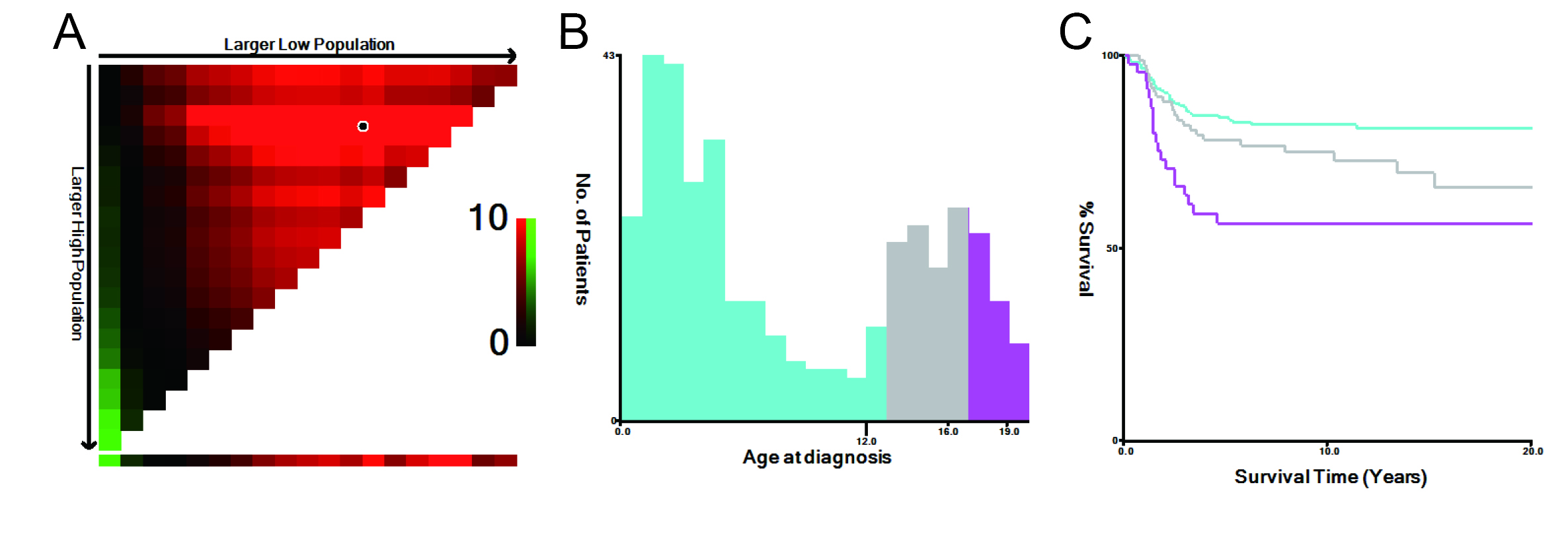

Supplement: Supplementary Figure 1 — The X-tile program was exploited to define the optimal cut-point of age at diagnosis. (A) The graph shows that the optimal cut-point was identified as 13 and 16 years old. (B) Distribution histogram of three groups divided by two cut-point in the overall cohort. (C) Significant differences between groups were evaluated using Kaplan-Meier's analysis (P < 0.05). [file Image_1.JPEG]
